# In this month’s Bulletin

**DOI:** 10.2471/BLT.18.000118

**Published:** 2018-01-01

In the editorial section, Ritu Sadana et al. (2) argue for the inclusion of older people in universal health coverage policies. Ramesh Krishnamurthy and Jason Hatton (3) describe how satellite imaging and other space technology can provide data for public health purposes. 

Jan Dirk Herberman (6) reports on measures taken to protect people and the environment from mercury exposure in Ghana, Malaysia and Thailand. Tom Frieden explains to Fiona Fleck (8) how concentrating on a few public health interventions to reduce noncommunicable diseases can help countries save the most lives.

## Haiti 

### How much should it cost to deliver services?

Ryan K McBain et al. (10–17) apply an activity-based costing exercise.

## India

### Who pays too much for health care?

Anamika Pandey et al. (18–28) track trends in catastrophic health expenditure over two decades.

## Philippines, Uganda

### When trials are not enough

Jennifer F Friedman et al. (59–65) identify barriers to treatment of schistosomiasis during pregnancy.

## Rwanda 

### New treatment requires a new response

Aimable Mbituyumuremyi et al. (51–58) propose a framework for the national response for Hepatitis C infections.

## Global

### Gender-based violence

Karel Blondeel et al. (29–41) review evidence of violent acts motivated by perceptions of sexual orientation and gender identity.

### Taking a life-course approach to health 

Shyama Kuruvilla et al. (42–50) look for ways to use the sustainable development goals.

### Challenges ahead

Effy Vayena et al. (66–68) explore policy implications of big data for the health sector.

### Preventing HIV transmission

Benjamin H Chi et al. (69–71) summarize attempts to engage both parents during the childbearing year.

